# Improved Test for Sugar in Urine

**Published:** 1875-01

**Authors:** W. S. Haines


Improved Test for Sugar in the Urine—*By W. S. Haines, M.D.* * * * Take of sulphate of copper, thirty grains; of hydrate of potassium (in sticks), one and a half drachms; of *pure* glycerine, two fluid drachms; and of pure water (distilled is best), six fluid ounces. Divide the water into two nearly equal portions; in one of these parts dissolve the sulphate of copper and the glycerine, and in the other portion the hydrate of potassium. Mix the two solutions and stir until any precipitate which may in the first place be formed is entirely re-dissolved. The test liquid thus prepared may now be poured into a bottle and set aside until needed.

* * * After a quantity of this solution has been prepared, testing for sugar in the urine becomes a matter of the greatest simplicity, and ought not to occasion the physician the slightest trouble. An ordinary sized test tube is to be filled to the depth of half an inch or so with the test liquid, and heat from any convenient source applied till it boils, when no change whatever should ensue. Now, two or three drops of the suspected urine are to be added, the test tube shaken slightly, so as to cause a mixture of the two fluids, and heat once more applied. If sugar is present in any considerable quantity, an abundant precipitate of the yellowish red sub-oxide of copper (cuprous oxide) will be produced, and this, remaining for a while suspended in the liquid, will make the latter appear almost opaque, by reflected light. If, however, the test solution undergoes no change on thus treating it with two or three drops of the urine on examination, we may be certain that no considerable quantity of sugar is present; but, in order to demostrate its *entire* absence, it will be necessary to continue the addition of the urine until a quantity has been added equal to half the bulk of the test liquid, and then apply heat once more. If any sugar whatever be present, it will now show itself by causing a characteristic precipitate of the sub-oxide of copper—it may be, however, only in small quantity. But if, on the contrary, sugar be entirely absent, no change in the test liquid will ensue, except a lightening of the color from the solution, and the appearance of a white, flocculent deposit of phosphates floating about in the light, greenish blue liquid. Care should be taken not to mistake this phosphatic deposit for one of the sub-oxide of copper; the former is white and flocculent, the latter yellowish red, and shows no disposition to form flocculi.—*Medical Examiner.*

